# Tree species determine soil microbial diversity: variation in fungal and bacterial communities in temperate forests

**DOI:** 10.1038/s41598-026-41297-6

**Published:** 2026-02-25

**Authors:** Wojciech Piaszczyk, Jarosław Lasota, Kacper Foremnik, Ewa Błońska

**Affiliations:** 1 Department of Ecology and Silviculture, Faculty of Forestry, University of Agriculture in Krakow, 29 Listopada 46 Str, 31-425 Krakow, Poland; 2 Department of Forest Biodiversity, Faculty of Forestry, University of Agriculture in Krakow, 29 Listopada 46 Str, 31-425 Krakow, Poland

**Keywords:** Microbial ecology, Soil biota, Temperate forest soils, NGS (next-generation sequencing), Soil–plant interactions, Ecology, Ecology, Environmental sciences, Microbiology

## Abstract

This study investigates the influence of three deciduous tree species: small-leaved linden (*Tilia cordata*), common beech (*Fagus sylvatica*), and sessile oak (*Quercus petraea*) on soil microbial diversity in temperate forest ecosystems. Conducted on loess soils in southern Poland, the research clarifies species-specific effects on soil microbiota and chemical properties, providing insights into tree-microbe-soil interactions in forest environments. Soil samples were collected from monospecific stands and analysed using next-generation sequencing (NGS). Fungal and bacterial DNA was extracted, and libraries targeting the ITS1 (fungi) and 16 S rRNA V3–V4 (bacteria) regions were sequenced using the Illumina MiSeq platform. Microbial communities were evaluated in relation to soil pH, nutrient content, and exchangeable cations. Linden soils had the highest pH (5.1–7.0) and calcium content (18.9 cmol(+)·kg⁻¹), while beech soils were the most acidic (pH 3.8–5.7) with the lowest calcium (8.0 cmol(+)·kg⁻¹). Fungal communities were dominated by Basidiomycota, Ascomycota, and Mortierellomycota, with varying proportions across species. Bacterial diversity was highest in linden and oak stands. Dominant bacterial phyla included Actinobacteriota, Proteobacteria, and Acidobacteriota. Each tree species hosted a distinct microbial community, reflecting its impact on soil properties and microbial structure. Tree species significantly shape soil microbial diversity and chemistry. Incorporating microbial data into forest management may enhance soil function, biodiversity conservation, and ecosystem resilience. Broader spatial sampling is recommended to generalize findings.

**Supplementary Information:**

The online version contains supplementary material available at 10.1038/s41598-026-41297-6.

## Introduction

Forests are complex ecosystems in which tree species play a pivotal role in determining the characteristics of the soil in which they grow^1–3^. Trees primarily influence soil properties through their litter inputs and root exudates, which regulate the quantity and quality of soil organic matter, thereby shaping the activity and composition of soil microbial communities^4^. Through the decomposition and transformation of plant-derived organic matter by microbes, nutrients are mineralised and made available for plant uptake, thereby driving nutrient cycling in forest soils. At the same time, plant roots and organic matter inputs contribute to soil structure formation and the development of physicochemical properties, such as aggregate stability and cation exchange capacity, mainly via their effects on organic matter accumulation and interactions with mineral particles^5^. Soil microorganisms, including fungi and bacteria, play a pivotal role in decomposition, nutrient mobilisation, and symbiotic interactions with plants. This links biological processes to soil’s chemical and physical properties^6,7^.

Tree species contribute different types of organic matter to the soil, which in turn determines the composition and activity of microbial communities^8^. Broadleaf species such as beech or oak typically produce nutrient-rich litter that decomposes more readily than conifer litter, influencing the abundance and diversity of soil microorganisms^9^. Conversely, species with recalcitrant litter may favour microbial communities adapted to slower decomposition processes^10^. Trees influence soil properties not only through the litter they produce but also via their root systems and associated exudates^11,12^. Root exudates also vary between tree species, shaping the microbial communities in the rhizosphere - a zone of intense microbial activity near roots^4^. However, the extent to which these microbial communities feed back to influence soil properties remains poorly understood.

Fungal and bacterial communities in forest soils are highly sensitive to tree-induced changes in soil conditions^13,14^. Fungi, particularly mycorrhizal species, establish mutualistic relationships with trees, enhancing nutrient uptake while benefiting from photosynthetically derived carbohydrates^15^. Bacteria, on the other hand, contribute to processes such as nitrogen fixation and organic matter decomposition^16^. Despite the growing body of research on forest microbiomes, there is still limited comparative data on how different broadleaved tree species shape soil chemical properties and associated microbial communities at the local scale^17,18^. This study examines how species-specific differences in soil chemistry relate to microbial community structure in linden, beech and oak stands. Our focus is on the dominant bacterial and fungal phyla and their distribution patterns in relation to key soil parameters, including pH, carbon and nitrogen content, and base cation concentrations. Linden trees are widespread across Europe, occurring in both natural forests and urban green spaces^19^. However, their influence on soil biological properties has received considerably less attention than that of beech or oak. Combining next-generation sequencing with detailed soil chemical analyses provides a comparative assessment of tree-driven microbial differentiation in temperate forest soils. The selected species - linden, beech and oak are key components of Central European forests, and are becoming increasingly relevant in the context of climate change, due to their contrasting ecological strategies and adaptive potential^20,21^. Linden has been shown in previous studies to exert a pronounced effect on soil chemical properties, including pH regulation, base cation availability and litter quality, all of which are key drivers of microbial community structure^22^. However, despite this recognised influence, current knowledge remains fragmented, as most studies have focused on selected soil parameters rather than providing an integrated view of microbial diversity and community composition. Although a basic impact of linden on soil processes has been established, it is still unclear to what extent this tree species shapes soil microbial communities in comparison with other dominant broadleaved species such as beech and oak. Our aim is to quantify the influence of these tree species on basic soil chemical properties and the diversity and composition of soil microbial communities, thereby contributing to a better understanding of tree-soil-microbe interactions in temperate forest ecosystems. The following research hypotheses were tested with the aim of understanding how different tree species influence soil microbial communities through changes in soil chemical properties, thereby shedding light on the ecological mechanisms shaping belowground biodiversity in temperate forests: (1) compared to the other tree species studied, linden has a more beneficial effect on soil chemical properties, particularly by increasing pH and Ca²⁺ content; (2) deciduous tree species such as beech, linden, and oak differentially affect soil microbial diversity, with linden promoting higher bacterial and fungal diversity; (3) linden supports a greater number of distinct bacterial taxa in soil compared to the other tree species; (4) each of the tree species studied supports a characteristic microbial community, suggesting species-specific influences on soil microbiota. These hypotheses aim to clarify not only whether microbial communities differ under different tree species, but also to explore the underlying soil conditions that may drive these differences, with implications for forest management and biodiversity conservation. This knowledge can inform strategies to conserve soil biodiversity, improve forest productivity and mitigate the effects of climate change.

## Materials and methods

### Study sites and data collection

**Fig. 1:**
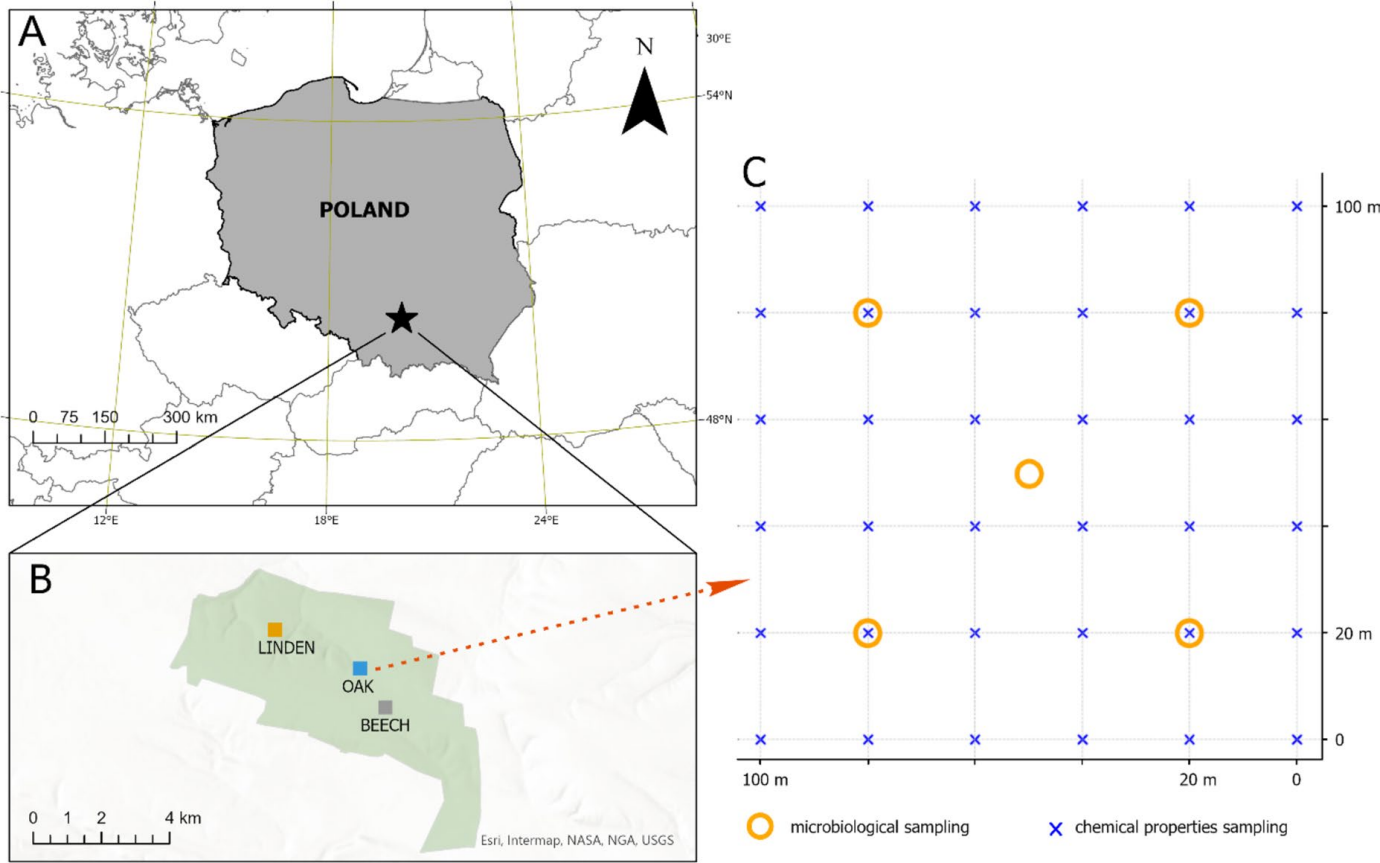
The location of research plots. (**A**) Location within Poland, (**B**) location within the forest complex, (**C**) soil sampling scheme within the research plot.

Three stands of similar age, around 80 years old, were selected for analysis: small-leaved linden (*Tilia cordata*), common beech (Fagus sylvatica) and sessile oak (*Quercus petraea*) (Fig. 1). The study plots were located within the administrative area of the Miechów Forest District in southern Poland (50.4795 N, 20.1363 E). Soil sampling was carried out with the permission of the Regional Directorate for Environmental Protection in Kraków, under permit number OP.6205.34.2022.MBa, issued on 31.05.2022. The study plots were located on Luvisols^23^, which developed on loess-covered wind-deposited silty sediments and are characterised by high porosity, good water retention and a rich mineral composition, consisting mainly of quartz, feldspars and carbonates. These soils are highly fertile but prone to erosion. All areas were characterised by similar geology, grain size and similar land use history. The tested soils were characterised by a silt grain size distribution (sand 25%, silt 66%, clay 9%), as determined using laser diffraction (Analysette 22, Fritsch, Idar-Oberstein, Germany). The study area had no history of agricultural use. Three study plots (each measuring 100 × 100 m) were established, one in each forest stand. Each plot was covered by a regular grid of 36 sampling points, 20 m apart. This systematic sampling design enabled us to capture spatial variability within each stand and obtain representative soil samples for comparison between tree species. Soil samples were collected from the topsoil to a depth of 15 cm at each sampling point. Thirty-six soil samples were taken from each study plot assigned to a specific tree species. By using a regular sampling grid, we aimed to minimise the influence of external factors such as climate and geological variability, and ensure that the observed differences were primarily due to biological processes associated with the tree species. In addition, the forest stands studied were in close proximity and had similar site conditions, further reducing the influence of unrelated environmental variability. Samples were placed in polythene bags and each bag was labelled with the name of the tree species and a sequential number. For microbiological analyses, including fungal and bacterial diversity, five additional soil samples were taken from each experimental plot (Fig. 1). For microbiological analyses, five independent samples were collected, each representing a composite sample formed from five subsamples that were thoroughly mixed and homogenized. Samples intended for microbiological analysis were transported to the laboratory in insulated containers on ice, and were transferred to a -80 °C freezer within a few hours of being collected. All fieldwork was carried out in September 2022.

### Chemical properties

The soil properties of all 36 individual samples per plot were determined using standard pedological methods^24,25^. The soil pH was measured in both H_2_O and KCl in accordance with PN-ISO 10,390^26^. Carbon (C) and nitrogen (N) contents were measured using an elemental analyser (LECO CNS TrueMac Analyzer, Leco, St. Joseph, MI, USA). Base cation (Ca²⁺, Mg²⁺, K⁺, Na⁺) concentrations were determined after extracting 10 g of soil with 100 mL of 1 M NH₄Cl solution. The extracts were subsequently analyzed using inductively coupled plasma-optical emission spectrometry (ICP-OES, iCAP 6500 DUO, Thermo Fisher Scientific, Cambridge, UK).

### Fungal and bacterial DNA library preparation from the soil

DNA was isolated from 1 g of soil according to the Genomic Mini AX Bacteria+ protocol (A&A Biotechnology, Poland). Mechanical lysis was performed using zirconia balls in a FastPrep-24 homogeniser. In addition, lyticase (A&A Biotechnology, Poland) was used for enzymatic lysis. Fungal DNA libraries were prepared for the ITS1 rDNA region amplified with ITS1F^27^. Bacterial DNA libraries were prepared for the V3-V4 16 S rDNA region amplified with 341 F and 785R primers^28^. PCR was performed in a reaction mixture containing 15 ng genomic DNA using Q5 Hot Start High-Fidelity 2X Master Mix (New England Biolabs, USA). Indexing PCR was performed using the Nextera XT Index Kit (Illumina). The PCR was performed using NEBNext^®^ High-Fidelity 2X PCR Master Mix (M0541) according to the manufacturer’s protocol, which is publicly available on the producer’s website. After indexing, samples were purified using AMPure XP beads and verified using a Bioanalyzer (Agilent Technologies, US) and qPCR. DNA libraries were sequenced on the Illumina MiSeq platform (2 × 300 bp paired-end) by Genomed (Poland). Sequencing depth was 50,000 reads per sample.

NGS data for fungi were processed using QIIME^29^. Samples were demultiplexed and fastq files were generated using MiSeq Reporter v 2.6 (Illumina). Adaptor and low quality sequences (below Q20) were removed using cutadapt^30^. Paired sequences were joined using the seqprep algorithm. Usearch61 was used for chimera removal^31^. Fungal reads were clustered using the uclust algorithm^31^ and BLASTed against ITS sequences UNITE v8.2^32,33^. Operational taxonomic units (OTUs) were filtered for very low abundance, and only OTUs with a relative abundance of at least 0.01% were used for further analysis.

Bacterial NGS data were processed in QIIME^29^. Demultiplexing and FASTQ file generation were performed with MiSeq Reporter v2.6 (Illumina), followed by quality and adapter trimming using cutadapt^30^. Sequence inference, paired-end merging, and chimera removal were conducted using the DADA2 algorithm^34^. Reads were checked against 16 S rRNA sequences from the Silva 138 database^35^. Bacterial amplicon sequence variants (ASVs) were processed and analysed as described above for fungal OTUs.

### Statistical analysis

Statistical analysis was performed using the statistical software R^36^ and R Studio^37^. The packages readxl, dplyr, scales and ggplot2 were used to generate plots of relative abundance of bacterial and fungal phyla. The heatmap package was used to generate heat maps. Fungal species occurrence data were used to calculate the relative abundance of fungal species and to generate an abundance heat map (including Euclidean cluster analysis) using log10(x + 1) transformed abundance data for fungal species whose total number exceeded 500 reads for at least five plots with a given tree species in the Illumina metabarcode. All statistical analyses and visualisations were performed using R and its associated packages. Normality was tested using the Shapiro–Wilk test, while Levene’s test was employed to verify variance homogeneity.The Kruskal-Wallis test was used to indicate differences in soil characteristics between the studied tree species. Pearson correlation coefficient was used to evaluate the relationships between soil characteristics. Principal component analysis (PCA) analysis was performed to demonstrate the relationships between dominant microbial groups and soil properties. We sought to determine which soil properties were key to specific microbial groups. We also included tree species in the PCA analysis. Principal Component Analysis (PCA) was performed using the MASS, factoextra, and ggfortify packages in R, based on Euclidean distances. The significance threshold was set at *p* < 0.05. Significant differences in soil properties (Kruskal-Wallis, *p* < 0.05) were observed, with large effect sizes (η² > 0.3) for pH and Ca²⁺. Confidence intervals were calculated where applicable to provide robust estimates of variability. Fungal functional guilds were assigned using the FUNGuild database (v1.1) based on genus-level taxonomic assignments, as species-level identification was not consistently available across all taxa^38^. Only taxa with “Probable” or “Highly Probable” confidence rankings were included in downstream functional analyses to reduce uncertainty in functional classification. Relative abundances of functional guilds were calculated per sample by summing taxon abundances and expressing them as percentages of the total fungal community. For visualization and comparative analyses, functional assignments were summarized into major trophic modes (saprotrophs, symbiotrophs, pathotrophs and others). All analyses were performed in R^36^ and R Studio^37^.

## Results

### Chemical properties

**Fig. 2:**
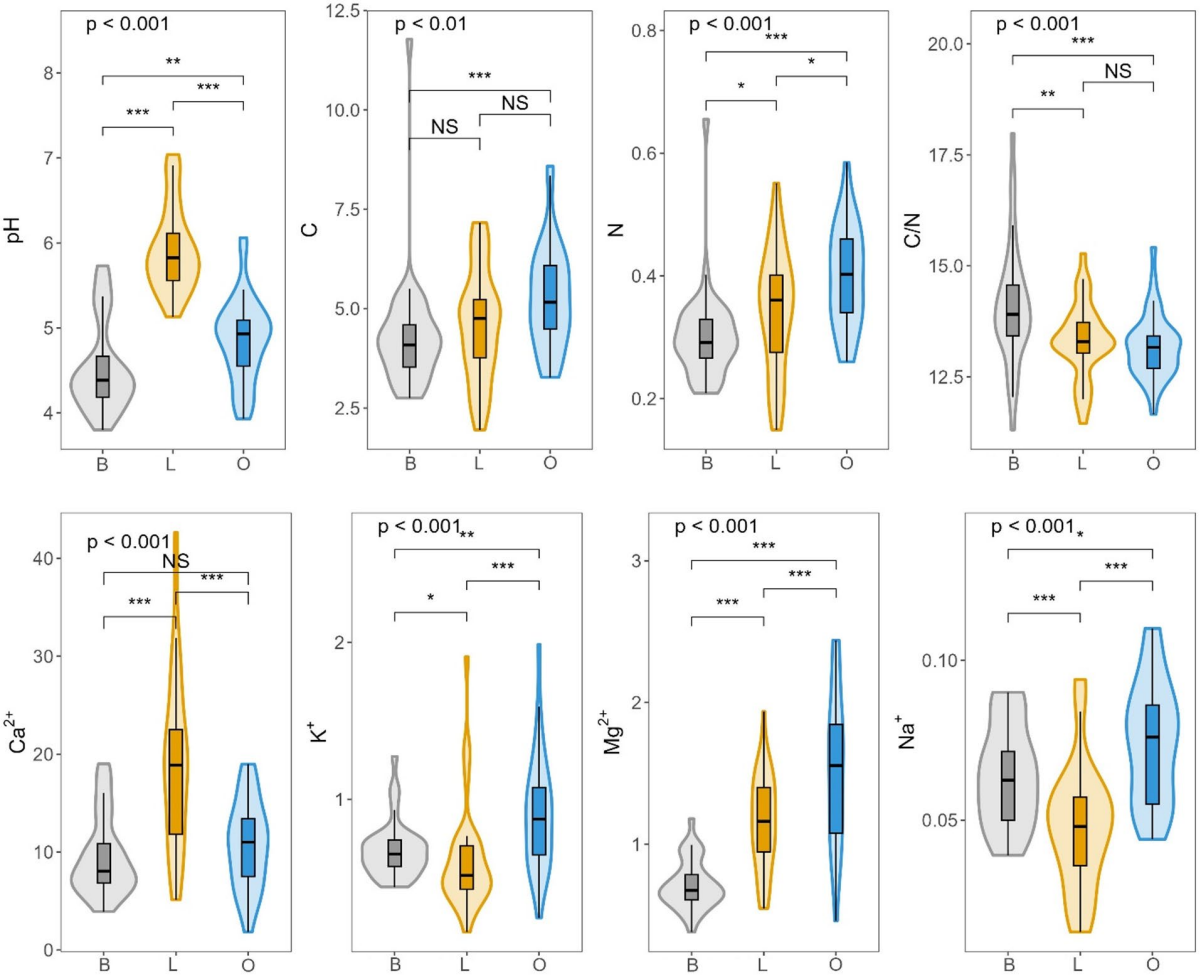
Variation in soil chemical properties under different tree species: beech (B), linden (L), and oak (O). Violin plots represent the distribution of values, with embedded boxplots indicating the median and interquartile range. Differences among species were evaluated using the Kruskal-Wallis test (*n* = 108), with global p-values reported above each panel. Pairwise differences were assessed using the Wilcoxon rank-sum test. Significance levels: ns = not significant, * *p* < 0.05, ** *p* < 0.01, *** *p* < 0.001. Units: C (%), N (%), Ca²⁺, K⁺, Mg²⁺, Na⁺ (all in cmol(+)·kg⁻¹).

The chemical properties of the soil differed between the linden, beech and oak stands, with significant contrasts observed among the three forest types (Fig. 2). The soil in the linden stand had the highest pH values (5.1-7.0), indicating its more alkaline nature, while the soil under the beech was the most acidic (3.8–5.7). The soil in the oak stand was characterised by intermediate pH values (5.5-6.0). The analysis of the organic carbon (C) and nitrogen (N) content revealed significant differences between the tree stands. The highest values were observed in oak soils, while beech soils contained significantly lower amounts of both elements. The C/N ratio was highest in beech soils, while linden and oak soils had comparable but slightly lower ratios. Similarly, cation concentrations (Ca²⁺, Mg²⁺, K⁺, Na⁺) varied significantly between stands. Linden soils had the highest calcium content, whereas beech soils had the lowest calcium and magnesium concentrations, corresponding to their more acidic conditions. Oak soils had the highest concentrations of magnesium, potassium and sodium. Potassium and sodium showed greater variability between stands, with the lowest values recorded in linden soils (Fig. 2).

**Fig. 3:**
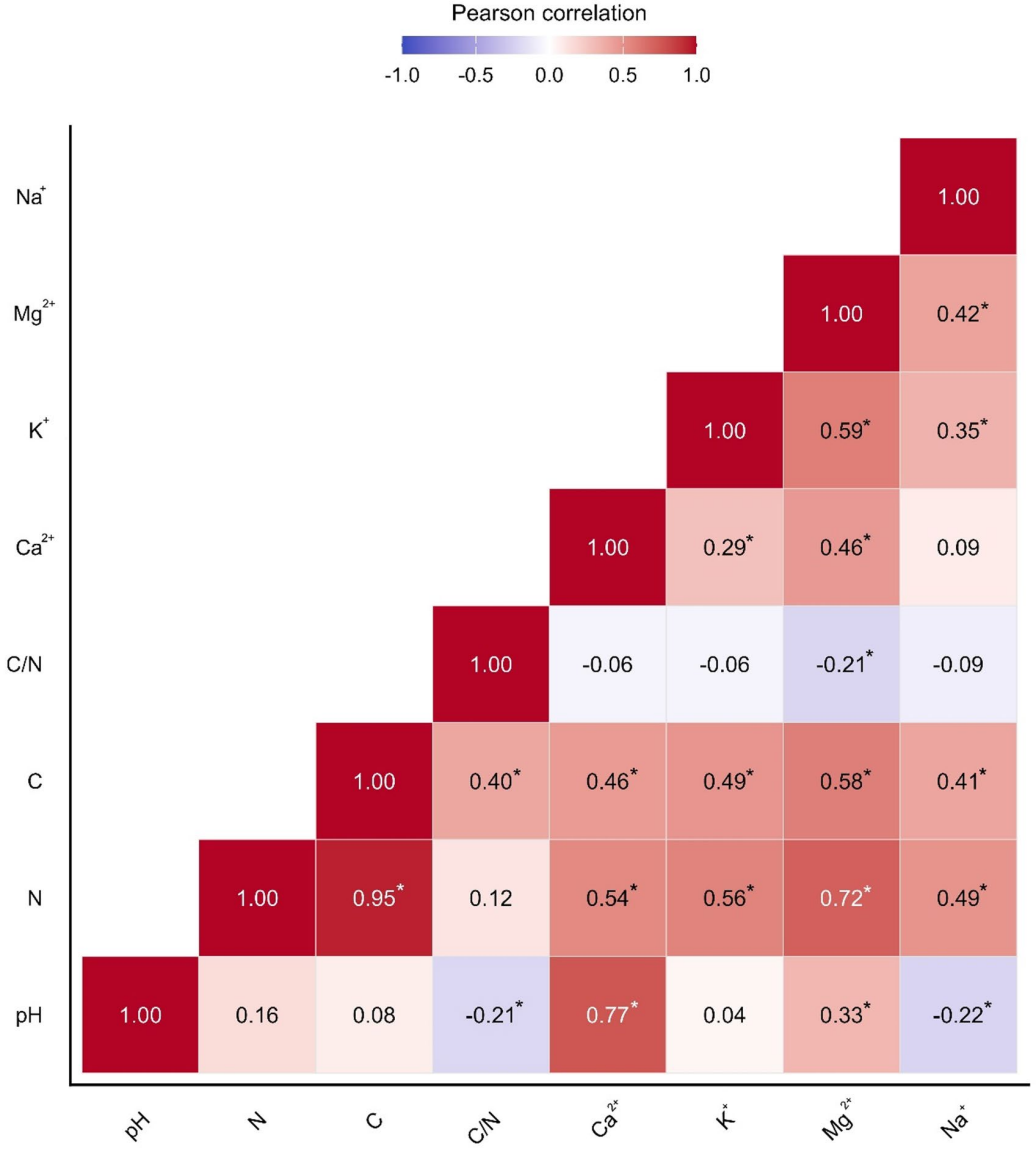
Pearson correlation coefficients between the soil properties tested. The values in the cells indicate the strength and direction of the correlations. Statistically significant correlations (*p* < 0.05) are indicated by asterisks.

Soils with a higher pH value were characterised by higher concentrations of exchangeable Ca²⁺ and Mg²⁺ ions. In contrast, organic carbon and nitrogen content varied together across the samples. Conversely, higher pH values were associated with lower C/N ratios (Fig. 3).

### Fungal and bacterial diversity

There were marked differences in the abundance and diversity of soil microorganisms between the analysed stand types. In all stand types the average number of fungal genera was similar, with 251 genera in the soil of oak stands, 255 in the soil of linden stands and 266 genera in the soil of beech stands (Figure S1). Beech plots were the most diverse in this respect (the number of fungal genera ranged from 245 to 282). The range of fungal genera for oak and linden plots was somewhat narrower, with 239–266 and 235–266, respectively (Figure S1). The number of bacterial genera found in the soil of beech stands was significantly lower (217–312, average 288) (Figure S2). The soils of oak and linden stands were richer in terms of the number of bacterial genera, with an average of 350 genera for oak (range 337–358) and 357 genera for linden (range 342–382) (Figure S2).

**Fig. 4:**
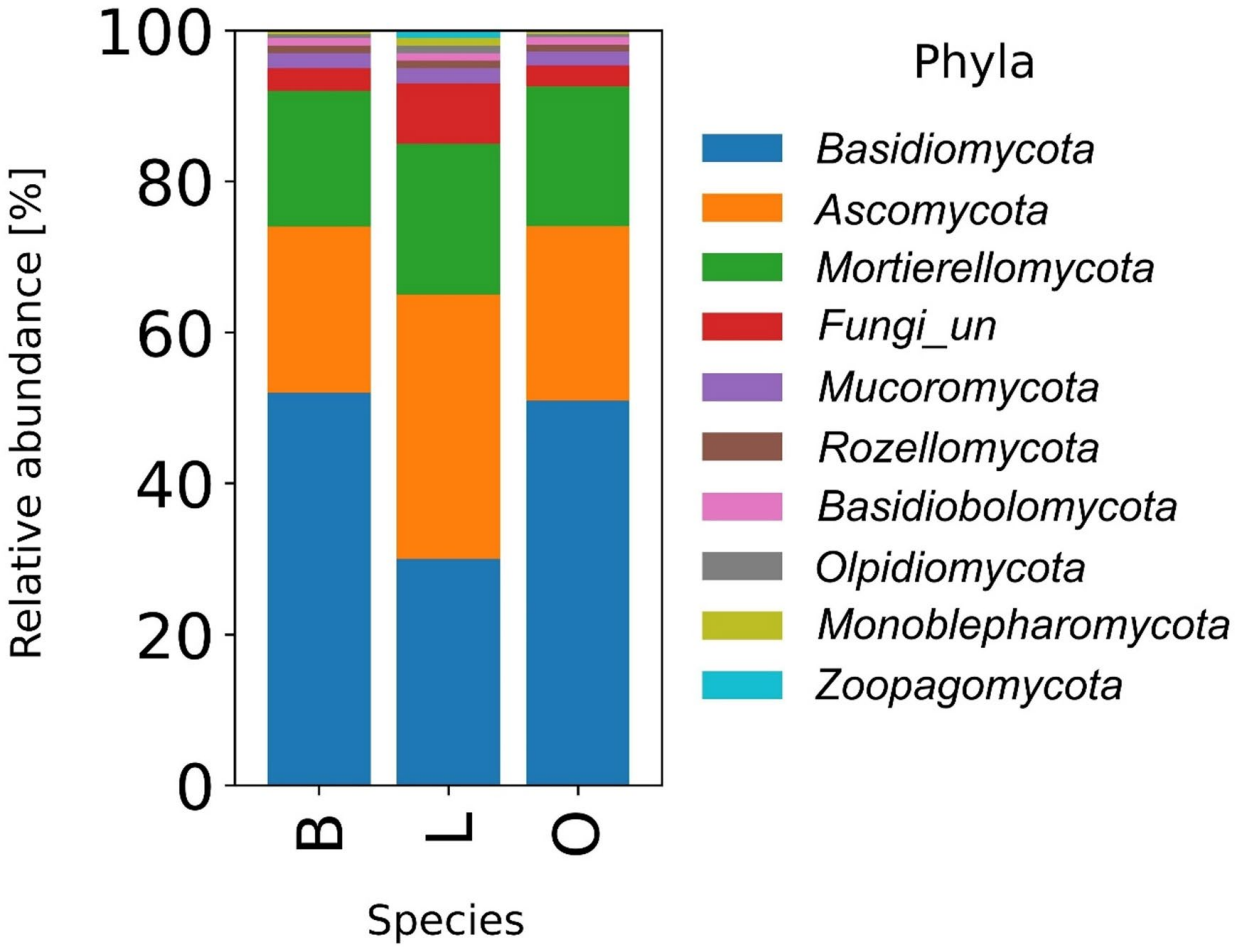
Relative abundance of fungal phyla identified in soil under influence of different trees species (B – beech, L – lime, O – oak).

**Fig. 5:**
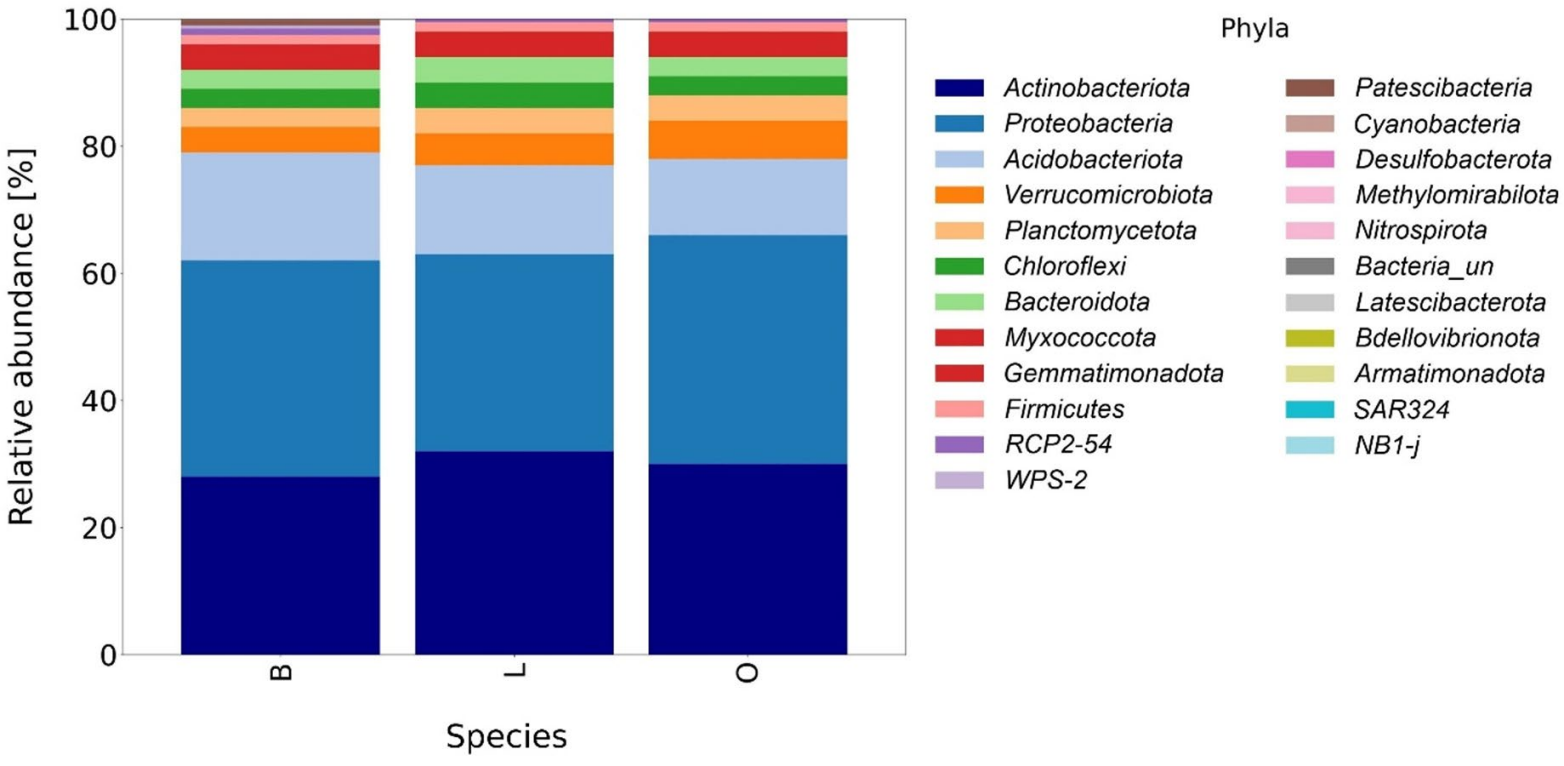
Relative abundance of bacterial phyla identified in soil under influence of different trees species (B – beech, L – lime, O – oak).

The overall microbial composition varied between stand types (Figs. 4 and 5). Dominant fungal phyla included *Basidiomycota*, *Ascomycota* and *Mortierellomycota*, with proportions varying between tree species. In particular, the linden plots had a higher proportion of *Ascomycota* and unidentified fungi, whereas the beech and oak plots were dominated by *Basidiomycota*. Minor fungal phyla such as *Mucoromycota* and *Rozellomycota* were slightly more abundant in the soil of beech stands (Fig. 4).

Among the bacteria, the main clusters were *Actinobacteriota*, *Proteobacteria* and *Acidobacteriota*. Beech plots had more *Acidobacteriota*, while oak and linden plots had more *Actinobacteriota*. Some smaller clusters, such as *Verrucomicrobiota* and *Myxococcota*, varied between tree species. Other clusters showed specific enrichments, e.g. *Planctomycetota* in oak, *Chloroflexi* in linden, and *Bacteroidota* in both oak and linden plots (Fig. 5).

At the genus level, soil of beech stands was dominated by *Mortierella*, *Russula* and *Lactarius*, with several genera present in low abundance but significantly more abundant than in the other stands (Figure S3). Soils of oak stands showed a high abundance of *Russula* and *Mortierella*, with a distinct set of genera such as *Penicillium*, *Tuber* and *Amanita*. The linden plots also had *Mortierella* as a dominant genus, with a uniquely diverse set of fungal genera that were more abundant or exclusive compared to the other stands.

**Fig. 6:**
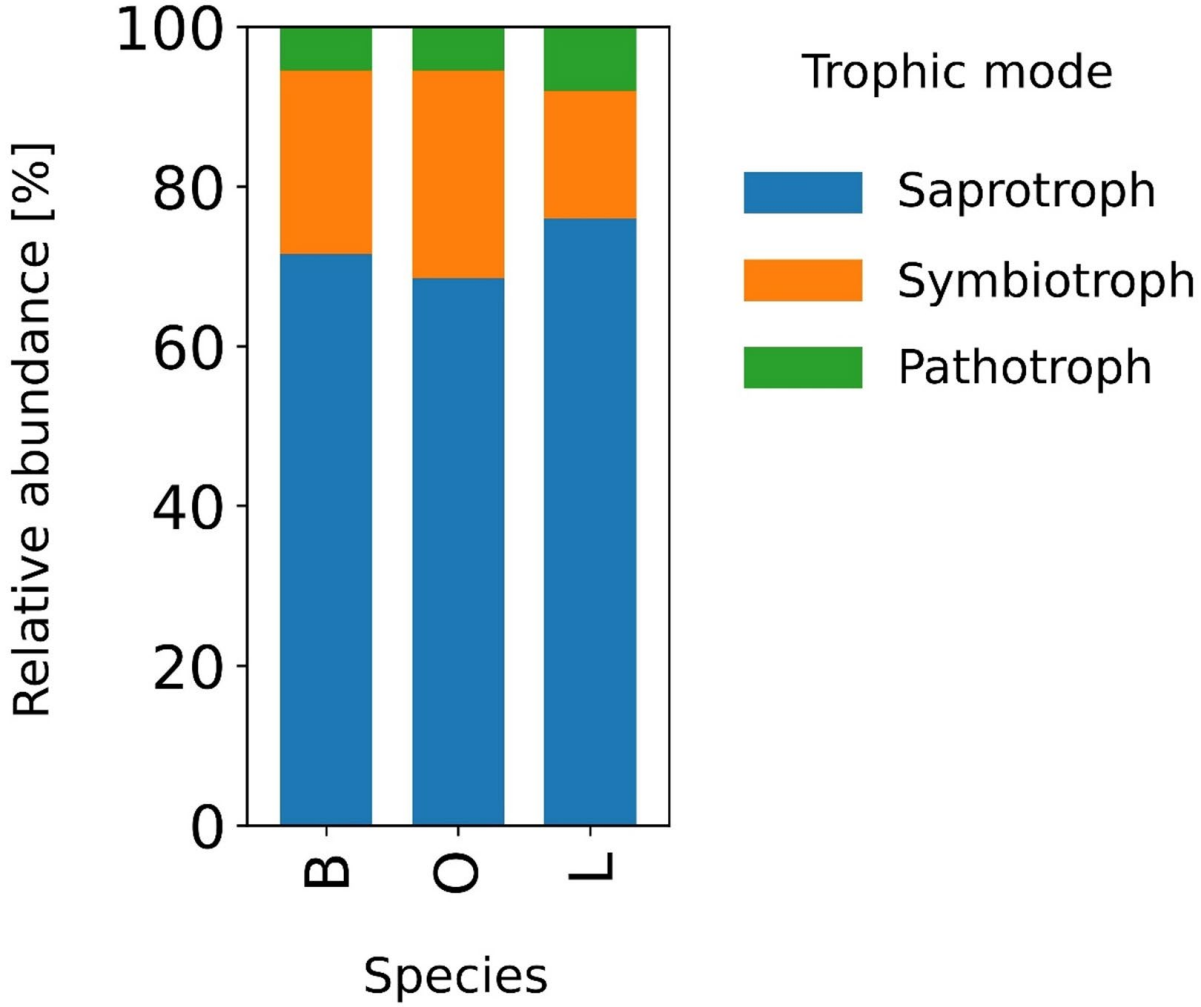
Relative abundance of fungal functional guilds identified in soil under influence of different trees species (B – beech, L – lime, O – oak).

Bacterial diversity was high in all plots, but no single genus dominated (Figure S4). In beech plots, *Acidothermus* and members of *Gaiellales* and *Elsterales* were most abundant. Oak plots shared some of these genera but differed in relative proportions. Soils of linden stands had a greater diversity of less abundant genera, including some taxa exclusive to this stand type, such as *Pedomicrobium* and *Ilumatobacter*. This microbial composition not only highlights taxonomic diversity, but also suggests ecological differentiation between the stands. Future studies should investigate functional aspects of these microbial communities. The analysis of the relative abundance of fungal functional guilds revealed clear differences in their structure among samples collected beneath beech (B), oak (O), and linden (L) trees (Fig. 6). In all analyzed samples, saprotrophs constituted the dominant group, accounting for approximately 68% to 78% of the total fungal abundance. The highest proportion of saprotrophs was observed in samples collected beneath linden (L), where their relative abundance was consistently high and exceeded 75% in most samples. In samples collected beneath beech (B), the proportion of saprotrophs was slightly lower and more uniform, ranging from approximately 70% to 72%. Samples collected beneath oak (O) were characterized by an intermediate proportion of this guild, accounting for approximately 68% to 70%. The second most abundant group consisted of symbiotrophs. Their highest relative abundance was recorded in samples collected beneath oak (O), where they accounted for approximately 26% to 27% of the fungal community. In samples collected beneath beech (B), the proportion of symbiotrophs was slightly lower, at approximately 23% to 24%. The lowest relative abundance of symbiotrophs was observed in samples collected beneath linden (L), where their contribution ranged from approximately 13% to 17%. Pathotrophs represented the least abundant functional guild across all analyzed variants. Their relative abundance was low and relatively stable, ranging from approximately 4% to 6% in samples collected beneath beech and oak, and from approximately 6% to 8% in samples collected beneath linden.

### Associations between microbial communities and soil chemical properties

**Fig. 7:**
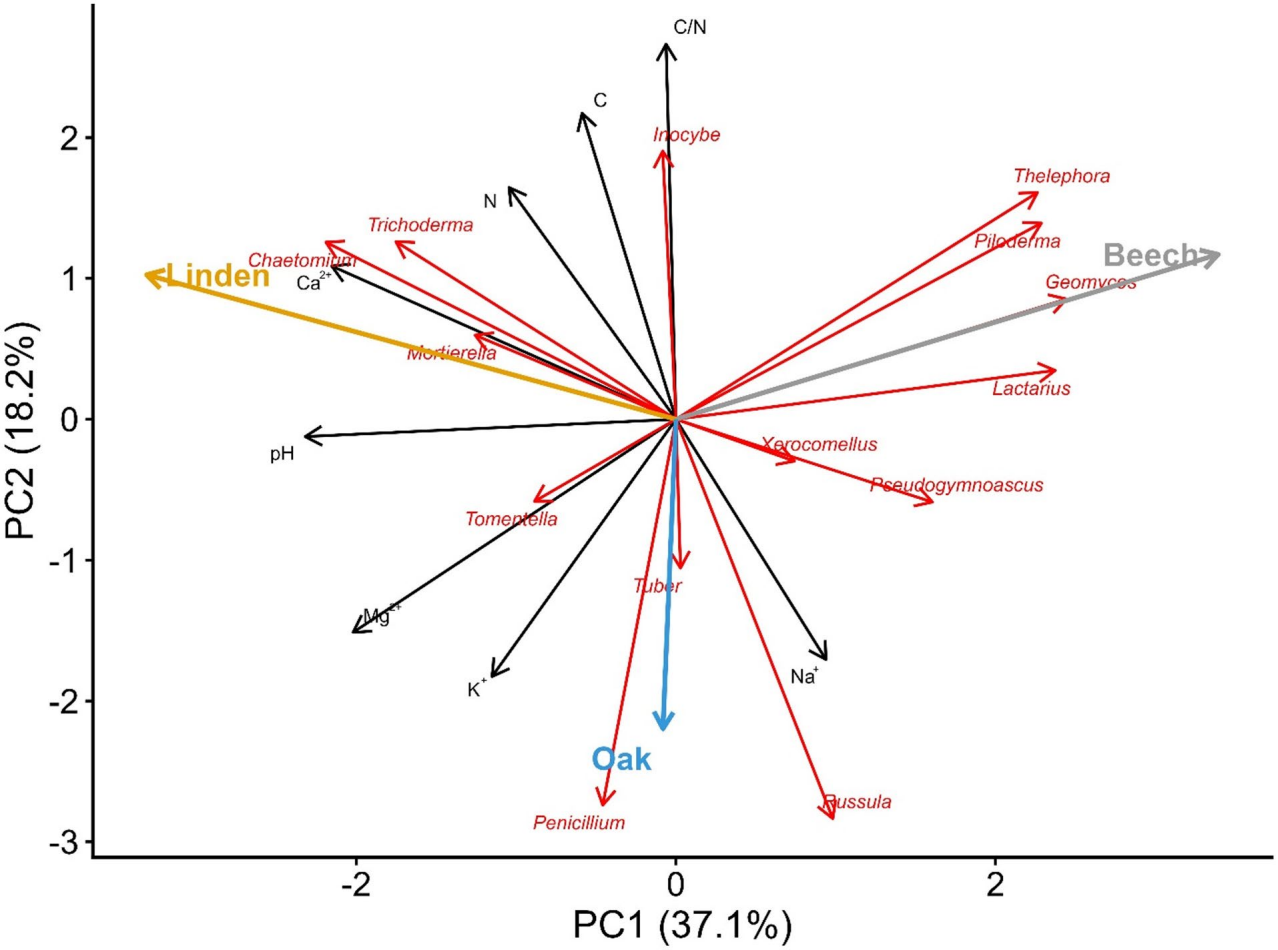
Principal component analysis (PCA) showing relationships between soil chemical properties, dominant fungal phyla (14 most abundant) and the three stands studied.

Principal component analysis (PCA) shows the relationships between dominant microbial groups and basic soil chemical properties. Vectors indicate the strength and direction of correlations between environmental variables and microbial structure. For fungi, genera such as *Chaetomium*, *Trichoderma* and *Mortierella* were strongly associated with higher soil calcium and pH. *Lactarius*, *Geomyces*, *Piloderma* and *Telephora* were associated with lower pH. *Inocybe* correlated with higher carbon content, nitrogen content and C/N ratio, while *Russula*, *Penicillium*, *Tomentella* and *Tuber* showed associations with potassium, sodium and magnesium content (Fig. 7).

**Fig. 8:**
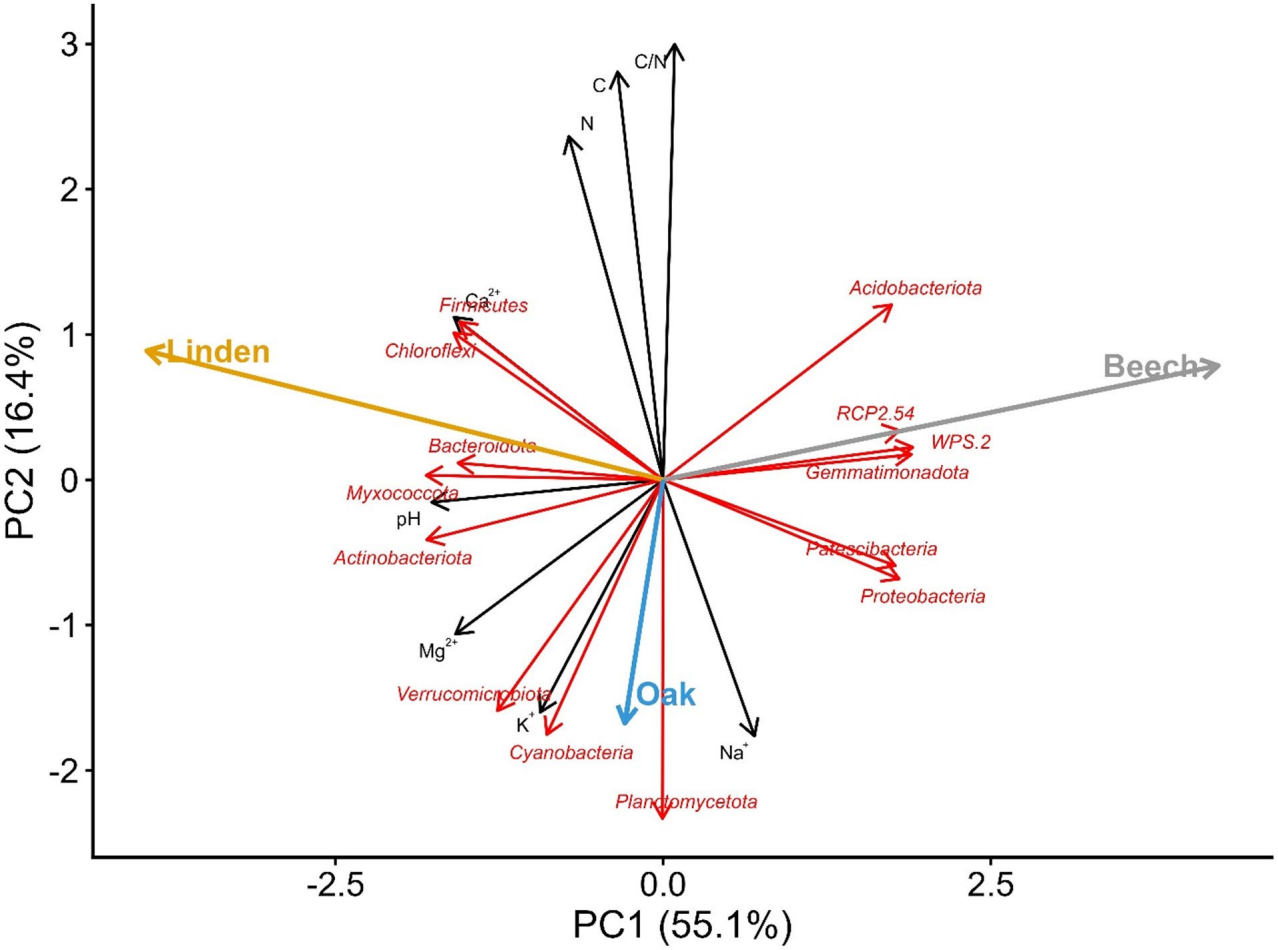
Principal component analysis (PCA) showing relationships between soil chemical properties, dominant bacterial phyla (14 most abundant) and the three stands studied.

Similar relationships were found for bacteria. Phyla such as *Actinobacteriota*, *Firmicutes*, *Chloroflexi*, *Myxococcota* and *Bacteroidota* were strongly associated with higher pH and calcium and magnesium content. *Acidobacteriota*, *Gemmatimonadota*, *RCP2.54* and *WPS-2* were associated with lower pH, while *Planctomycetota*, *Verrucomicrobiota* and *Cyanobacteria* showed an association with increased sodium and potassium levels (Fig. 8).

## Discussion

Our study revealed substantial differences in the soil microbiomes associated with stands of beech, oak, and linden. Among these, linden had the strongest impact, fostering the development of distinct fungal communities and supporting a richer and more diverse bacterial community in the soil. This difference in microbial groups with linden is the result of different properties, especially pH and the amount of base cations.

In our study, under conditions of soil and climate homogeneity, the differences in microbial structure found are the result of interactions between different tree species. The studied tree species significantly influenced key soil chemical properties, including pH, C, N, and exchangeable cations. Linden stands, in particular, had higher pH, elevated calcium content, and a lower C/N ratio, indicating a strong impact on soil chemistry. This finding suggests that organic matter in these soils is subject to more efficient decomposition processes, probably driven by favourable microbial conditions associated with linden trees, consistent with recent evidence that tree species locally regulate decomposition dynamics through species-specific soil conditioning^39^. The reduced C/N ratio indicates improved nutrient cycling and greater availability of nitrogen for plant uptake, further emphasising the beneficial role of linden trees in improving soil fertility.

As this study focused on the topsoil layer (0–15 cm) sampled in late summer, it does not capture vertical or seasonal variation in microbial communities. This should be addressed in future studies^40^. Although lime stands were characterised by higher pH, elevated calcium concentrations and a relatively low C/N ratio, it should be emphasised that soils under oak also exhibited comparable or even higher absolute contents of organic carbon and nitrogen. This indicates that oak stands may promote substantial organic matter accumulation despite less pronounced changes in soil pH. The relatively low C/N ratio observed under oak, together with intermediate pH values, suggests that decomposition and nutrient turnover processes in these soils are also efficient, albeit potentially driven by different mechanisms than those prevailing under lime.

The study presented here is not the first to compare the effects of different tree species on soil environmental characteristics. Beech, oak and linden have already been compared in a common garden trial conducted in central Poland^41,42^. These earlier findings provide valuable context for interpreting the results of the present study. A particularly favourable effect on the accumulation of soil organic matter was found in the soils of the linden plots, which was confirmed by the stock of carbon bound in the light fraction occurring in occlusions with mineral particles (POM fraction) and the so-called heavy fraction strongly bound to the mineral fraction (MAF fraction). The beneficial effect of linden on the soil environment was also confirmed by the finding of high activity of enzymes involved in the cycling of C, N and P. In the soil of linden plots, β-glucosidase and phosphatase activities were found to be high compared to oak and beech stands, N-acetyl-β-glucosaminidase activity was also high especially compared to that found in the soil of beech plots^41^. These enzyme activities, considered together, indicate an increase in microbial-driven nutrient transformations in linden-associated soils.

For instance, Stefanowicz et al.^43^ found the highest total microbial biomass and G⁺ bacterial biomass in linden plots, while Reich et al.^44^ reported more earthworm biomass in linden and beech stands. In our study, we observed a greater richness of bacterial taxa in soils under linden and oak compared to beech, suggesting that the high enzymatic activity may be linked to increased microbial diversity and overall soil quality. Januszek et al.^45^ also confirmed the particularly favourable conditions in linden stands, reporting high activity of both intracellular enzymes (e.g. dehydrogenases, indicating total microbial activity) and extracellular enzymes (e.g. urease and proteases) involved in carbon and nitrogen cycling. Taken together, these findings suggest that the increased microbial activity in linden soils forms part of a wider trend towards improved biological soil function.

Key genera (e.g., *Mortierella*, *Chaetomium*) associated with linden soils enhance nutrient cycling and pathogen suppression, underscoring their functional importance^46^. In addition, more than a dozen fungal genera with abundances of 1–4% were found here, of which the genera with significantly higher abundances compared to the soils of the other two tree species were: *Tomentella*, *Chaetomium*, *Trichoderma*, *Plectosphaerella*, *Phallus*, *Cortinarius*, *Laetinaevia*, *Minimedusa* and *Neonectria*.

The observed differences in microbial community composition across tree species likely reflect distinct ecosystem functions driven by microbial taxa. For example, fungal genera such as *Trichoderma*,* Chaetomium*, and *Clonostachys*—more abundant in linden soils—are known for their roles in cellulose decomposition, and plant growth promotion, contributing to enhanced nutrient cycling and forest health. The presence of *Tomentella and Cortinarius*, ectomycorrhizal fungi, indicates enhanced nutrient uptake and tree-root symbioses, especially under linden and beech stands.

*Trichoderma* fungi are antagonists of soil pathogens through the production of secondary metabolites and hydrolytic enzymes such as chitinases and glucanases. They stimulate plant growth by inducing resistance and improving nutrient availability^47^. A number of the above-mentioned fungi associated with the soils of linden stands play a key role in maintaining favourable soil properties through the decomposition of organic matter, participation in biogeochemical cycles, and suppression of plant pathogens.

In addition, bacterial genera with low relative abundance such as *Amaricoccus*, *Terrabacter*, *Thermomonas*, *Pseudarthrobacter*, *Nannocystaceae*, *Microvirga*, *Flavihumibacter* and *Bosea* were found exclusively in the soils of linden stands. Many of these, including *Terrabacter* and *Flavihumibacter*, are saprotrophic bacteria capable of degrading complex organic compounds.

Symbiotic bacteria, such as *Microvirga*, form associations with plants, fixing atmospheric nitrogen and improving its availability to plants^48^. Antagonistic bacteria, such as *Nannocystaceae*, produce bioactive compounds that limit the growth of soil pathogens and promote plant health^49^. *Acidothermus* and *Solibacter*, more abundant in beech and oak stands, are linked to decomposition of complex organic matter under acidic and oligotrophic conditions, suggesting their role in carbon cycling. Contaminant degrading bacteria, such as *Thermomonas* and *Pseudarthrobacter*, in turn participate in bioremediation by removing toxins and persistent organic matter^50,51^. Overall, the presence of different tree species affects both the diversity of microbes in the soil and the functional potential of soil communities. This has implications for nutrient cycling and the resilience of forest ecosystems.

These differences in microbial functional potential, driven by tree species, are also reflected in the structure of fungal functional guilds. Saprotrophs dominated in all analyzed variants, but their relative abundance was highest in samples under lime trees and lowest in those under oak trees. This result is consistent with the concept that plant species characteristics, including the quality and rate of decomposition of organic precipitation, are key factors controlling decomposition processes and the structure of the soil microbiome^52,53^. The high proportion of saprotrophs, particularly in samples under lime trees, may indicate the dominance of organic matter decomposition processes and relatively rapid carbon cycling. Tree species with more easily degradable litter favor the development of saprotrophic organisms, which can lead to accelerated mineralization. The highest proportion of symbiotrophs recorded in samples under oak trees indicates the greater importance of mutualistic interactions, particularly mycorrhizal interactions, in this stand type. Deciduous trees such as oak and beech are strongly associated with ectomycorrhizal fungi, which play a key role in nutrient uptake and long-term soil carbon stabilization^54,55^. The lower relative abundance of symbiotrophs in samples from under the lime tree may reflect the lower importance of mycorrhiza in the functioning of this system or the predominance of other resource acquisition strategies by soil microorganisms. This phenomenon is consistent with the observed global functional variability of soil fungal communities, dependent on environmental conditions and vegetation composition^56^. Pathotrophs were the least abundant guild in all variants analyzed, suggesting that pathogenic pressure in the studied soils was relatively low. This result is typical for stable forest ecosystems, where diverse and functionally rich microbial communities can limit the expansion of pathogens through competition and biological suppression mechanisms^57^. As Nguyen et al.^58^ point out, the ecological function of microorganisms is not always clearly linked to their taxonomic position, and the actual functional activity may be strongly dependent on environmental conditions.

## Conclusions

The study reveals significant differences in fungal and bacterial diversity in soils under oak, linden, and beech forests. These findings underline the complexity of microbial communities and their potential functional implications for forest ecosystems. Deciduous tree species such as beech, linden, and oak influence soil microbial diversity in different ways, with linden supporting a higher diversity of fungi and bacteria compared to other species. The dominance of fungal groups such as *Basidiomycota*, *Ascomycota*, and *Mortierellomycota* was noted. Linden soils showed a higher proportion of *Ascomycota*. Bacterial diversity was highest in oak and linden soils. *Actinobacteriota*, *Proteobacteria*, and *Acidobacteriota* were dominant bacterial phyla, with notable differences in their relative abundance between forest types. Our findings support all proposed research hypotheses and underline the ecological importance of tree species selection. As linden promotes beneficial soil conditions and microbial diversity, it may be especially valuable in reforestation and biodiversity-oriented forest management. We recommend incorporating microbial indicators into forest soil monitoring and considering species like linden to enhance soil health and ecosystem services. Future studies should include multiple stands per species across diverse geographical locations to account for spatial variability and enhance generalizability.

## Supplementary Information

Below is the link to the electronic supplementary material.

Supplementary Material 1

Supplementary Material 2

Supplementary Material 3

Supplementary Material 4

Supplementary Material 5

## Data Availability

Libraries with the fungal sequences analysed have been deposited with the GenBank ( [https://www.ncbi.nlm.nih.gov/](https:/www.ncbi.nlm.nih.gov) ) under project number PRJNA1345375 ( [https://www.ncbi.nlm.nih.gov/bioproject/PRJNA1345375](https:/www.ncbi.nlm.nih.gov/bioproject/PRJNA1345375) ), and those with the bacterial sequence have been deposited under project number PRJNA1345340 ( [https://www.ncbi.nlm.nih.gov/bioproject/PRJNA1345340](https:/www.ncbi.nlm.nih.gov/bioproject/PRJNA1345340) ). The dataset supporting this study has been deposited in Zenodo and is available under DOI: 10.5281/zenodo.14832174.

## References

[1] Hobbie, S. E. et al. Tree species effects on soil organic matter dynamics: The role of soil cation composition. *Ecosystems* **10** (6), 999–1018. 10.1007/s10021-007-9073-4 (2007).

[2] Mueller, K. E. et al. Tree species effects on coupled cycles of carbon, nitrogen, and acidity in mineral soils at a common garden experiment. *Biogeochemistry* **111** (1), 601–614. 10.1007/s10533-011-9695-7 (2012).

[3] Adamczyk, B., Kilpeläinen, P., Kitunen, V. & Smolander, A. Potential activities of enzymes involved in N, C, P, and S cycling in boreal forest soil under different tree species. *Pedobiologia* **57** (2), 97–102. 10.1016/j.pedobi.2013.12.003 (2014).

[4] Staszel-Szlachta, K., Lasota, J., Szlachta, A. & Błońska, E. The impact of root systems and their exudates in different tree species on soil properties and microorganisms in temperate forest ecosystems. *BMC Plant. Biol.* **24** (1), 45. 10.1186/s12870-024-04724-2 (2024). 38212695 10.1186/s12870-024-04724-2 PMC10785385

[5] Paluch, J. & Gruba, P. Effect of local species composition on topsoil properties in mixed stands with silver fir (*Abies alba* Mill). *Forestry* **85** (3), 413–426. 10.1093/forestry/cps040 (2012).

[6] Wu, H. et al. Unveiling the crucial role of soil microorganisms in carbon cycling: A review. *Sci. Total Environ.* **909**, 168627. 10.1016/j.scitotenv.2023.168627 (2024). 37977383 10.1016/j.scitotenv.2023.168627

[7] Baldrian, P., López-Mondéjar, R. & Kohout, P. Forest microbiome and global change. *Nat. Rev. Microbiol.* **21** (8), 487–501. 10.1038/s41579-023-00876-4 (2023). 36941408 10.1038/s41579-023-00876-4

[8] Klimek, B., Chodak, M., Jaźwa, M. & Niklińska, M. Functional diversity of soil microbial communities in boreal and temperate Scots pine forests. *Eur. J. Res.* **135** (4), 731–742. 10.1007/s10342-016-0968-5 (2016).

[9] Prescott, E. C. Litter decomposition: What controls it and how can we alter it to sequester more carbon in forest soils? *Biogeochemistry* **101** (1), 133–149. 10.1007/s10533-010-9439-0 (2010).

[10] Błońska, E., Lasota, J. & Gruba, P. Effect of temperate forest tree species on soil dehydrogenase and urease activities in relation to other properties of soil derived from loess and glaciofluvial sand. *Ecol. Res.* **31** (5), 655–664. 10.1007/s11284-016-1375-6 (2016).

[11] Kaźmierczak, M., Błońska, E., Kempf, M., Zarek, M. & Lasota, J. Rhizosphere effect: Microbial and enzymatic dynamics in the rhizosphere of various shrub species. *Plant. Soil.* **511** (1), 245–262. 10.1007/s11104-024-06981-4 (2025).

[12] Lasota, J., Kaźmierczak, M. & Błońska, E. Understory shrub root systems and their exudates improve soil biochemistry in pine stands in temperate climate. *Rhizosphere* **29**, 100868. 10.1016/j.rhisph.2024.100868 (2024).

[13] Napoletano, P. et al. Quantifying the immediate response of soil to wild boar (*Sus scrofa* L.) grubbing in Mediterranean olive orchards. *Soil. Syst.* **7** (2), 38. 10.3390/soilsystems7020038 (2023).

[14] Rożek, K. et al. Soil fungal and bacterial community structure in monocultures of fourteen tree species of the temperate zone. *Ecol. Manage.* **530**, 120751. 10.1016/j.foreco.2022.120751 (2023).

[15] Hawkins, M. J. et al. Mycorrhizal mycelium as a global carbon pool. *Curr. Biol.* **33** (11), 560–573. 10.1016/j.cub.2023.02.027 (2023). 10.1016/j.cub.2023.02.027 37279689

[16] Rashid, M. I. et al. Bacteria and fungi can contribute to nutrients bioavailability and aggregate formation in degraded soils. *Microbiol. Res.* **183**, 26–41. 10.1016/j.micres.2015.11.007 (2016). 26805616 10.1016/j.micres.2015.11.007

[17] Wu, D. G., D’Amico, V. & Trammell, T. L. E. Soil bacterial communities in urban deciduous forests are filtered by site identity, soil chemistry, and shrub presence. *Sci. Rep.* **14** (1), 31735. 10.1038/s41598-024-81838-5 (2024). 39738340 10.1038/s41598-024-81838-5 PMC11686066

[18] Lasota, J., Ważny, R., Kaźmierczak, M. & Błońska, E. The effect of shrubs admixture in pine forest stands on soil bacterial and fungal communities and accumulation of polycyclic aromatic hydrocarbons. *Sci. Rep.* **13** (1), 16512. 10.1038/s41598-023-43925-x (2023). 37783867 10.1038/s41598-023-43925-x PMC10545714

[19] Weryszko-Chmielewska, E., Piotrowska-Weryszko, K. & Dąbrowska, A. Response of *Tilia* sp. L. to climate warming in urban conditions – Phenological and aerobiological studies. *Urban Urban Green.* **43**, 126369. 10.1016/j.ufug.2019.126369 (2019).

[20] Stobbe, A. & Gumnior, M. Palaeoecology as a tool for the future management of forest ecosystems in Hesse (Central Germany): Beech (*Fagus sylvatica* L.) versus lime (*Tilia cordata* Mill). *Forests* **12** (7), 924. 10.3390/f12070924 (2021).

[21] Fuchs, Z. et al. European beech (*Fagus sylvatica* L.): A promising candidate for future forest ecosystems in Central Europe amid climate change. *Cent. Eur. J.* **70** (2), 62–76. 10.2478/forj-2023-0020 (2024).

[22] Vesterdal, L., Schmidt, I. K., Callesen, I., Nilsson, L. O. & Gundersen, P. Carbon and nitrogen in forest floor and mineral soil under six common European tree species. *Ecol. Manage.* **255** (1), 35–48. 10.1016/j.foreco.2007.08.015 (2008).

[23] IUSS Working Group WRB. *World Reference Base for Soil Resources 2014: International Soil Classification System for Naming Soils and Creating Legends for Soil Maps. Update 2015. World Soil Resources Reports No. 106* (FAO, 2015).

[24] van Reeuwijk, L. P. (Ed.) *Procedures for Soil Analysis. Technical Paper 9* (ISRIC, 2002).

[25] Pansu, M. & Gautheyrou, J. *Handbook of Soil Analysis: Mineralogical, Organic and Inorganic Methods*. 10.1007/978-3-540-31211-6 (Springer, 2006).

[26] Polish Standard PN-ISO 10390. *Soil Quality—Determination of Soil pH* (1997).

[27] Gardes, M. & Bruns, T. D. ITS primers with enhanced specificity for basidiomycetes—Applications to the identification of mycorrhizae and rusts. *Mol. Ecol.* **2** (2), 113–118. 10.1111/j.1365-294X.1993.tb00005.x (1993). 8180733 10.1111/j.1365-294x.1993.tb00005.x

[28] Ferris, M. J., Muyzer, G. & Ward, D. M. Denaturing gradient gel electrophoresis profiles of 16S rRNA-defined populations inhabiting a hot spring microbial mat community. *Appl. Environ. Microbiol.* **62** (2), 340–346. 10.1128/aem.62.2.340-346.1996 (1996). 8593039 10.1128/aem.62.2.340-346.1996 PMC167804

[29] Caporaso, J. G. et al. QIIME allows analysis of high-throughput community sequencing data. *Nat. Methods*. **7** (5), 335–336. 10.1038/nmeth.f.303 (2010). 20383131 10.1038/nmeth.f.303 PMC3156573

[30] Martin, M. Cutadapt removes adapter sequences from high-throughput sequencing reads. *EMBnet J.* **17** (1), 10–12. 10.14806/ej.17.1.200 (2011).

[31] Edgar, R. C. Search and clustering orders of magnitude faster than BLAST. *Bioinformatics* **26** (19), 2460–2461. 10.1093/bioinformatics/btq461 (2010). 20709691 10.1093/bioinformatics/btq461

[32] Altschul, S. F., Gish, W., Miller, W., Myers, E. W. & Lipman, D. J. Basic local alignment search tool. *J. Mol. Biol.* **215** (3), 403–410. 10.1016/S0022-2836(05)80360-2 (1990). 2231712 10.1016/S0022-2836(05)80360-2

[33] Nilsson, R. H. et al. The UNITE database for molecular identification of fungi: Handling dark taxa and parallel taxonomic classifications. *Nucleic Acids Res.* **47** (D1), D259–D264. 10.1093/nar/gky1022 (2019). 30371820 10.1093/nar/gky1022 PMC6324048

[34] Callahan, B. J. et al. DADA2: High-resolution sample inference from Illumina amplicon data. *Nat. Methods*. **13** (7), 581–583. 10.1038/nmeth.3869 (2016). 27214047 10.1038/nmeth.3869 PMC4927377

[35] Quast, C. et al. The SILVA ribosomal RNA gene database project: Improved data processing and web-based tools. *Nucleic Acids Res.* **41** (D1), D590–D596. 10.1093/nar/gks1219 (2012). 23193283 10.1093/nar/gks1219 PMC3531112

[36] R Core Team. *R: A Language and Environment for Statistical Computing* (R Foundation for Statistical Computing, 2020).

[37] RStudio Team & RStudio *RStudio: Integrated Development for R* (PBC, 2020).

[38] Nguyen, N. H. et al. FUNGuild: An open annotation tool for parsing fungal community datasets by ecological guild. *Fungal Ecol.* **20**, 241–248. 10.1016/j.funeco.2015.06.006 (2016).

[39] Yates, C. et al. Temperate trees locally engineer decomposition and litter-bound microbiomes through differential litter deposits and species-specific soil conditioning. *New. Phytol*. **243** (3), 909–921. 10.1111/nph.19900 (2024). 38877705 10.1111/nph.19900

[40] Luo, X. et al. Soil horizons regulate bacterial community structure and functions in Dabie Mountain of East China. *Sci. Rep.* **13** (1), 15866. 10.1038/s41598-023-42981-7 (2023). 37739984 10.1038/s41598-023-42981-7 PMC10517015

[41] Błońska, E., Lasota, J., Prażuch, W. & Ilek, A. Stabilization of soil organic matter following the impact of selected tree species in temperate climate. *Catena* **243**, 108185. 10.1016/j.catena.2024.108185 (2024).

[42] Błońska, E., Lasota, J., Prażuch, W. & Ilek, A. Vertical variations in enzymatic activity and C:N:P stoichiometry in forest soils under the influence of different tree species. *Eur. J. Res.* **144** (1), 83–94. 10.1007/s10342-024-01742-5 (2025).

[43] Stefanowicz, A. M., Rożek, K., Stanek, M., Rola, K. & Zubek, S. Moderate effects of tree species identity on soil microbial communities and soil chemical properties in a common garden experiment. *Ecol. Manage.* **482**, 118799. 10.1016/j.foreco.2020.118799 (2021).

[44] Reich, P. B. et al. Linking litter calcium, earthworms and soil properties: A common garden test with 14 tree species. *Ecol. Lett.* **8** (8), 811–818. 10.1111/j.1461-0248.2005.00779.x (2005).

[45] Januszek, K., Lasota, J. & Fiślak, A. The evaluation of quality of the Carpathian lime tree forest and beech forests on the basis of some chemical and biochemical properties. *Acta Sci. Pol. Silv Colendar Rat. Ind. Lignar*. **5** (2), 71–87 (2006).

[46] Ning, Q. et al. Saprotrophic fungal communities in arable soils are strongly associated with soil fertility and stoichiometry. *Appl. Soil. Ecol.* **159**, 103843. 10.1016/j.apsoil.2020.103843 (2021).

[47] Harman, G. E., Howell, C. R., Viterbo, A., Chet, I. & Lorito, M. Trichoderma species—Opportunistic, avirulent plant symbionts. *Nat. Rev. Microbiol.* **2** (1), 43–56. 10.1038/nrmicro797 (2004). 15035008 10.1038/nrmicro797

[48] Ardley, J. K. et al. *Microvirga lupini* sp. nov., *Microvirga lotononidis* sp. nov. and *Microvirga zambiensis* sp. nov. are alphaproteobacterial root-nodule bacteria that specifically nodulate and fix nitrogen with geographically and taxonomically separate legume hosts. *Int. J. Syst. Evol. Microbiol.* **62** (11), 2579–2588. 10.1099/ijs.0.035097-0 (2012). 22199210 10.1099/ijs.0.035097-0

[49] Shimkets, L. J., Dworkin, M. & Reichenbach, H. The myxobacteria. *Prokaryotes* **7**, 31–115. 10.1007/0-387-30747-8_3 (2006).

[50] Busse, H. J. et al. *Thermomonas haemolytica* gen. nov., sp. nov., a gamma-proteobacterium from kaolin slurry. *Int. J. Syst. Evol. Microbiol.* **52** (2), 473–483. 10.1099/00207713-52-2-473 (2002). 11931159 10.1099/00207713-52-2-473

[51] Busse, H. J. Review of the taxonomy of the genus *Arthrobacter*, emendation of the genus *Arthrobacter* sensu lato, proposal to reclassify selected species of the genus *Arthrobacter* in the novel genera *Glutamicibacter* gen. nov., *Paeniglutamicibacter* gen. nov., *Pseudoglutamicibacter* gen. nov., *Paenarthrobacter* gen. nov. and *Pseudarthrobacter* gen. nov., and emended description of *Arthrobacter roseus*. *Int. J. Syst. Evol. Microbiol.* **66** (1), 9–37. 10.1099/ijsem.0.000702 (2016). 26486726 10.1099/ijsem.0.000702

[52] Cornwell, W. K. et al. Plant species traits are the predominant control on litter decomposition rates within biomes worldwide. *Ecol. Lett.* **11** (10), 1065–1071. 10.1111/j.1461-0248.2008.01219.x (2008). 18627410 10.1111/j.1461-0248.2008.01219.x

[53] Prescott, C. E. & Grayston, S. J. Tree species influence on microbial communities in litter and soil: Current knowledge and research needs. *Ecol. Manage.* **309**, 19–27. 10.1016/j.foreco.2013.02.034 (2013).

[54] Clemmensen, K. E. et al. Roots and associated fungi drive long-term carbon sequestration in boreal forest. *Science* **339** (6127), 1615–1618. 10.1126/science.1231923 (2013). 23539604 10.1126/science.1231923

[55] van der Heijden, M. G. A., Martin, F. M., Selosse, M. A. & Sanders, I. R. Mycorrhizal ecology and evolution: The past, the present, and the future. *New. Phytol*. **205** (4), 1406–1423. 10.1111/nph.13288 (2015). 25639293 10.1111/nph.13288

[56] Tedersoo, L. et al. Global diversity and geography of soil fungi. *Science* **346** (6213), 1256688. 10.1126/science.1256688 (2014). 25430773 10.1126/science.1256688

[57] Fierer, N. Embracing the unknown: Disentangling the complexities of the soil microbiome. *Nat. Rev. Microbiol.* **15** (10), 579–590. 10.1038/nrmicro.2017.87 (2017). 28824177 10.1038/nrmicro.2017.87

[58] Nguyen, D., Boberg, J., Ihrmark, K., Stenström, E. & Stenlid, J. Do foliar fungal communities of Norway spruce shift along a tree species diversity gradient in mature European forests? *Fungal Ecol.* **23**, 97–108 (2016).

