# Supplementary figures and images for: Tree species determine soil microbial diversity: variation in fungal and bacterial communities in temperate forests
Source: Sci Rep. 2026 Feb 25;16:11022. doi: 10.1038/s41598-026-41297-6 (PMC13043666; doi:10.1038/s41598-026-41297-6)

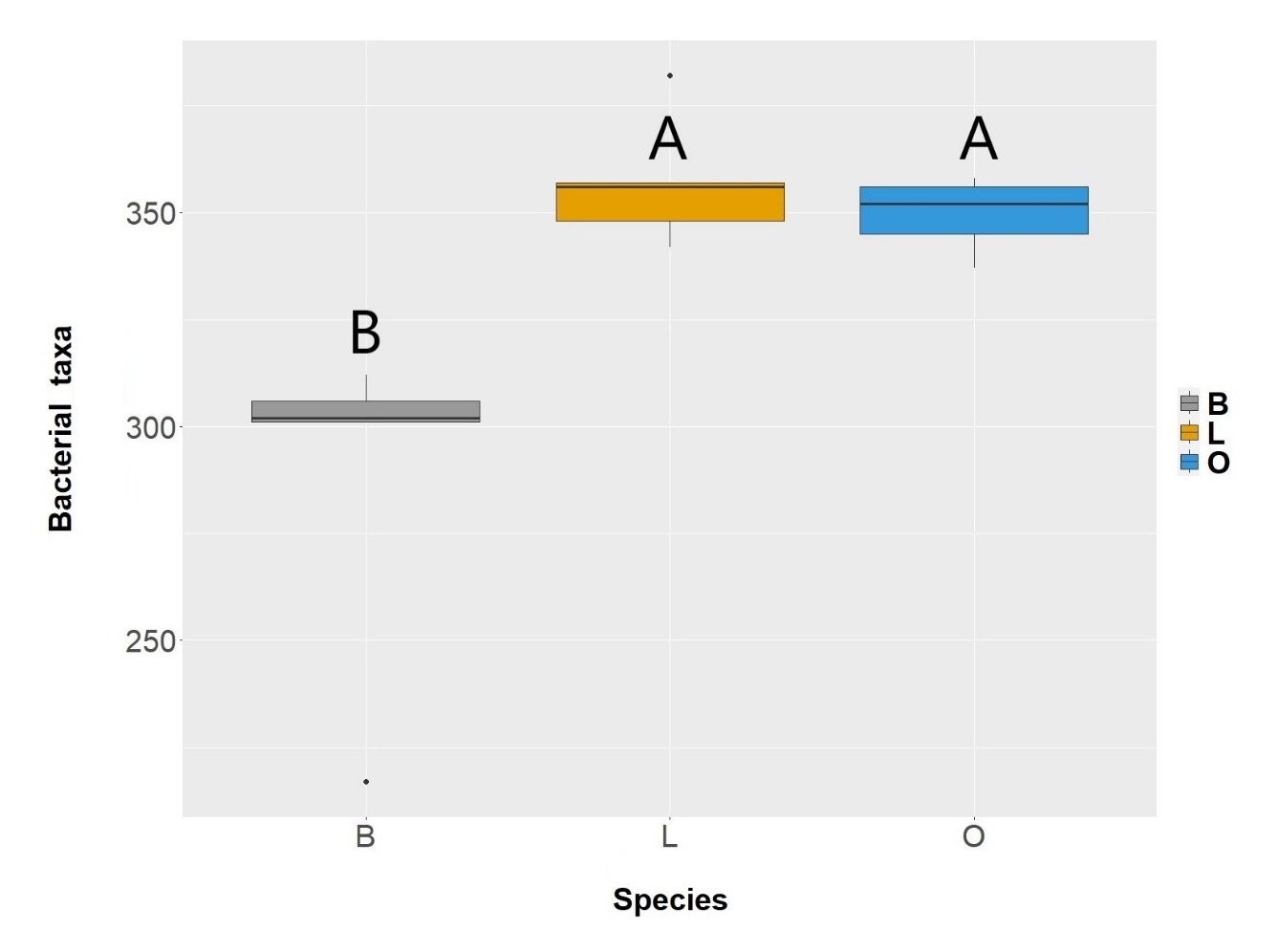

Supplement: Supplementary file 1 — Supplementary Material 1 [file 41598_2026_41297_MOESM1_ESM.jpg]

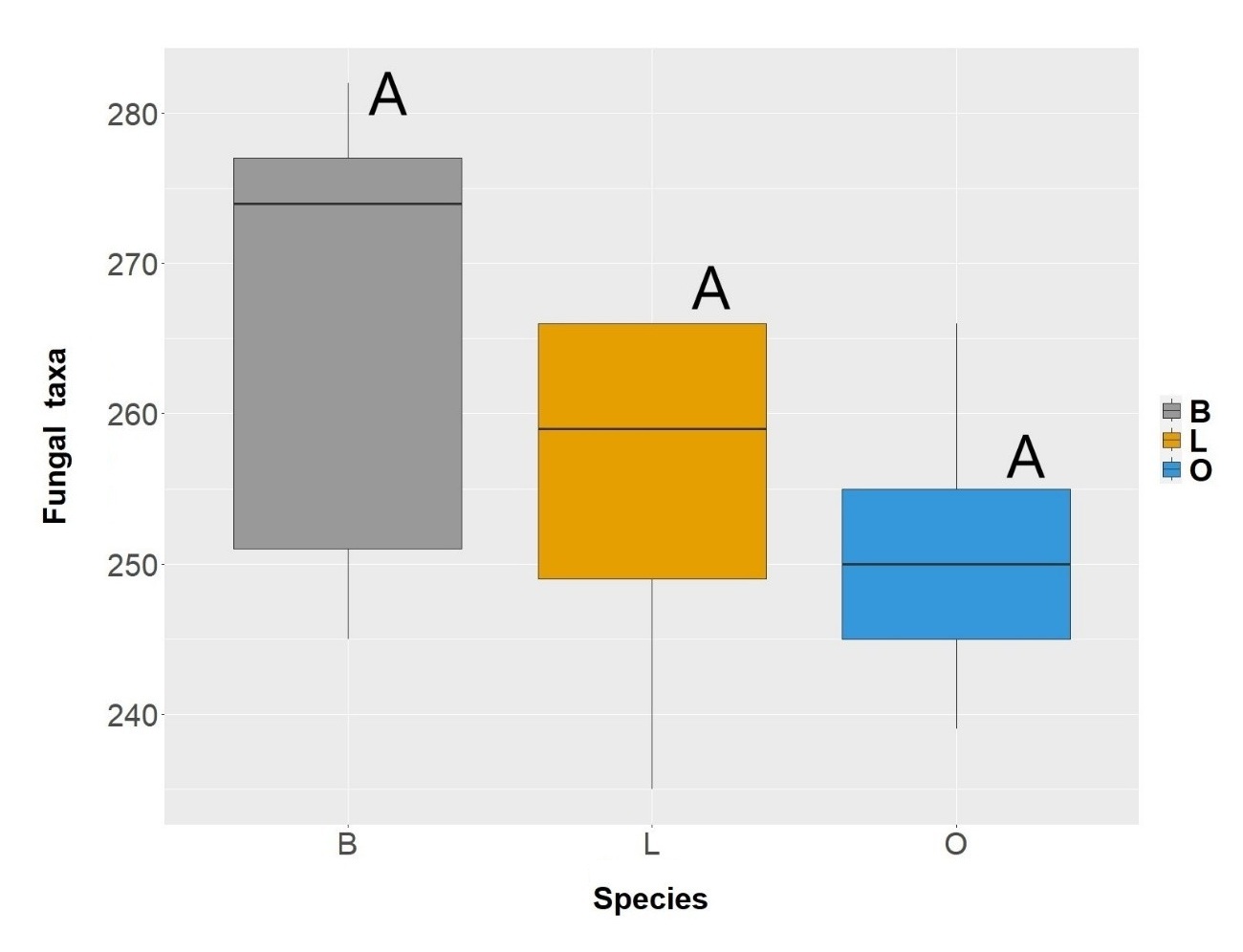

Supplement: Supplementary file 2 — Supplementary Material 2 [file 41598_2026_41297_MOESM2_ESM.jpg]

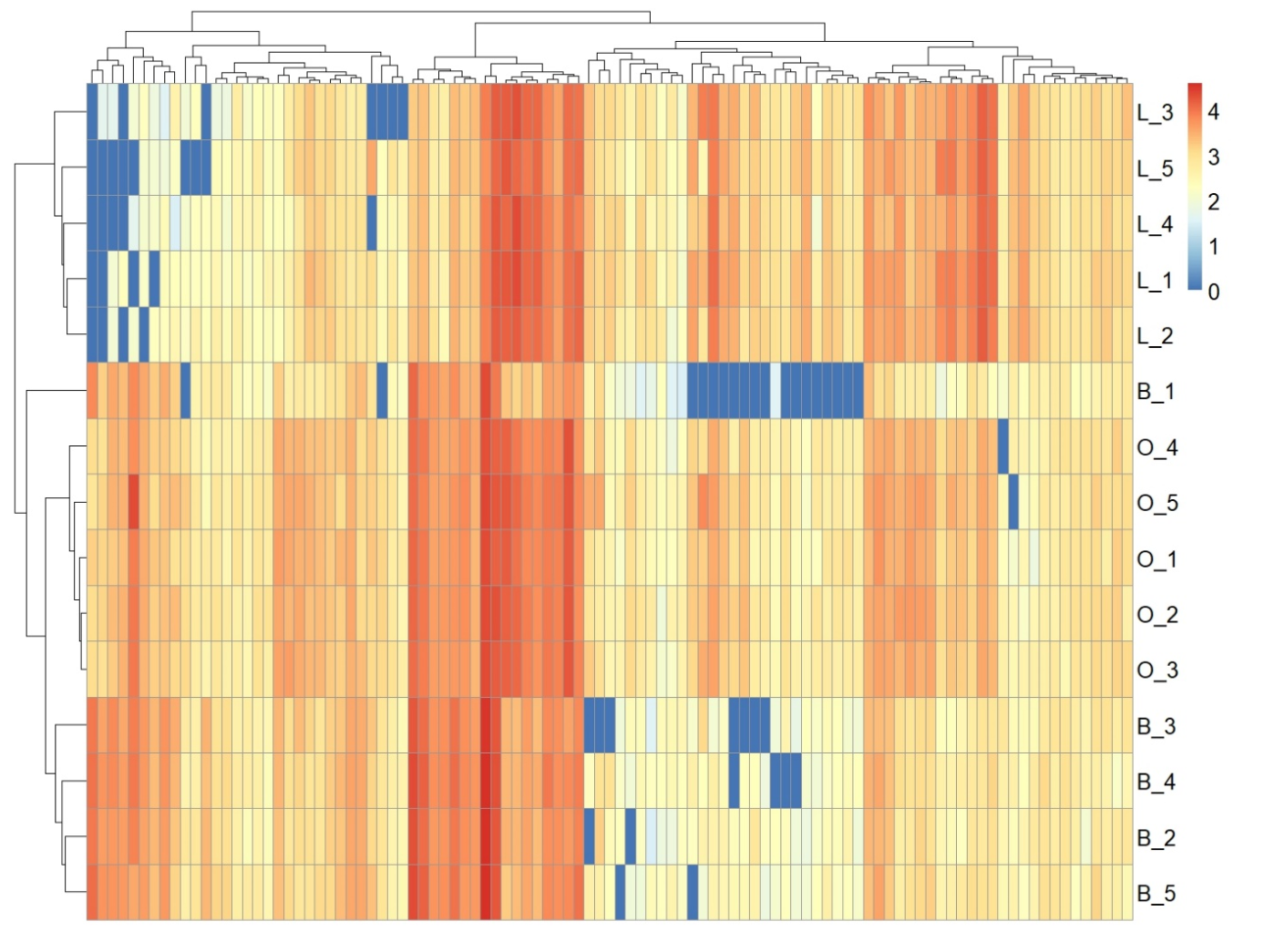

Supplement: Supplementary file 3 — Supplementary Material 3 [file 41598_2026_41297_MOESM3_ESM.png]

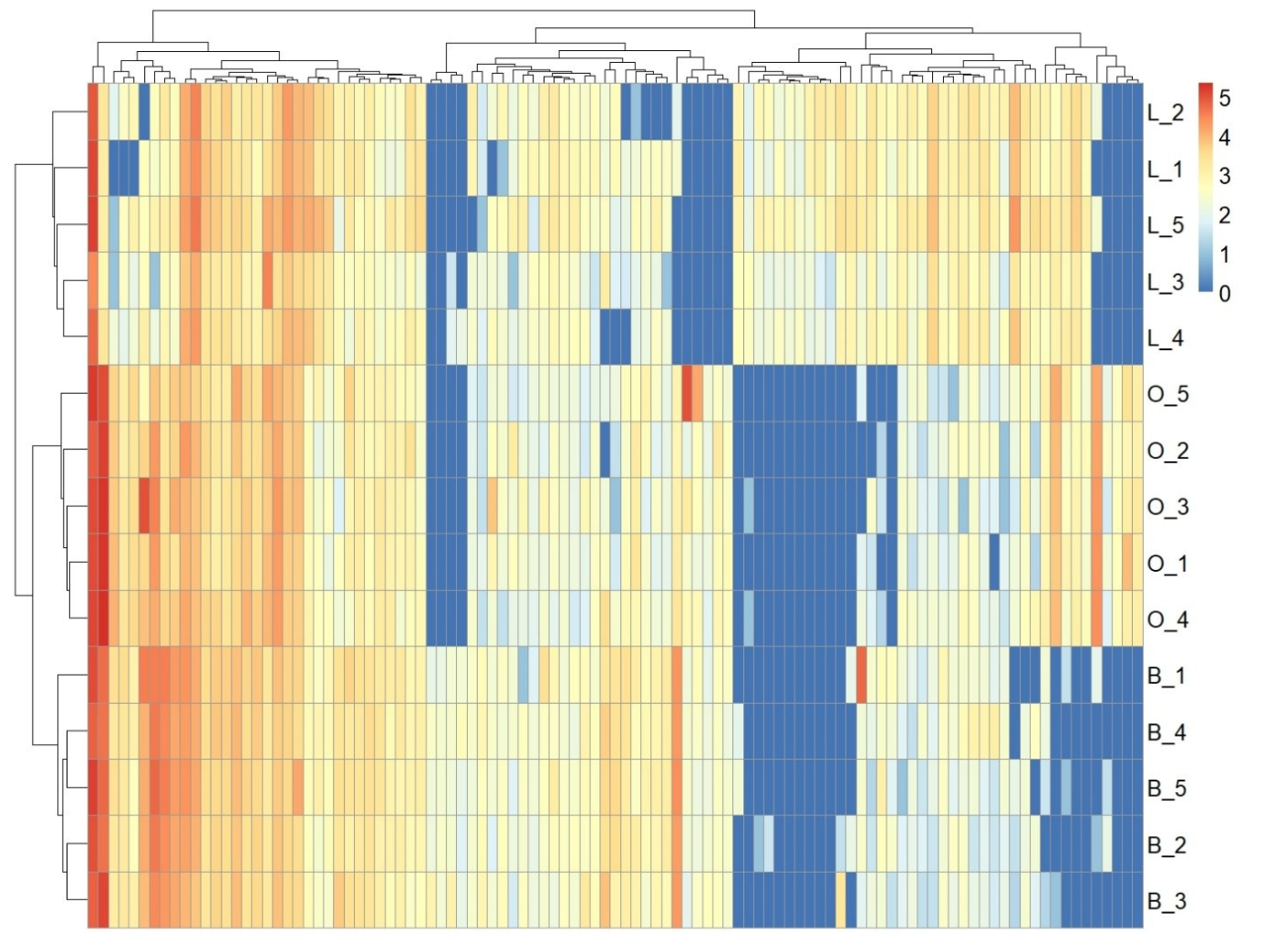

Supplement: Supplementary file 4 — Supplementary Material 4 [file 41598_2026_41297_MOESM4_ESM.png]
