# Supplementary material for: Tree species determine soil microbial diversity: variation in fungal and bacterial communities in temperate forests
Source: Sci Rep. 2026 Feb 25;16:11022. doi: 10.1038/s41598-026-41297-6 (PMC13043666; doi:10.1038/s41598-026-41297-6)
Supplement: Supplementary file 5 — Supplementary Material 5 [file 41598_2026_41297_MOESM5_ESM.docx]

*Figure_S1:* Number of fungal taxa identified in soil under influence of different trees species (B – beech, L – lime, O – oak; capital letters indicate differences between different species)

*Figure_S2:* Number of bacterial taxa identified in soil under influence of different trees species (B – beech, L – lime, O – oak; capital letters indicate differences between different species)

*Figure_S3*. Abundance heatmap based on the Illumina metabarcoding data constructed using log10 + 1 transformed abundance data for fungal species whose total number of reads exceeded 400 (B – beech, L – lime, O – oak)

***Figure_S4*.** Abundance heatmap based on the Illumina metabarcoding data constructed using log10 + 1 transformed abundance data for bacterial species whose total number of reads exceeded 400 (B – beech, L – lime, O – oak)
